# Occupational Exposure to Pesticides Among Farmworkers in Morocco: A Study Framework for Endocrine and Epigenetic Effect Assessment

**DOI:** 10.3390/toxics13050340

**Published:** 2025-04-25

**Authors:** Kaoutar Chbihi, Aziza Menouni, Imane Berni, Hala Chetouani, Said Abou-Said, Amal Amellah, Robin Lebegge, Eline Verscheure, Jeroen Vanoirbeek, Radu-Corneliu Duca, Lode Godderis, Samir El Jaafari

**Affiliations:** 1 Human Epidemiology and Environmental Health Team, Faculty of Sciences, Moulay Ismail University, Meknes 50000, Morocco; 2 Environment & Health Unit, Department of Public Health & Primary Care, Faculty of Medicine, Katholieke Universiteit Leuven, 3000 Leuven, Belgium; 3 Higher Institution of Nursing Professions and Health Techniques, Laâyoune 70000, Morocco; 4 Unit of Environmental Hygiene and Human Biological Monitoring, Laboratoire National de Santé (LNS), Department of Health Protection, L-3555 Dudelange, Luxembourg; 5 IDEWE, External Service for Prevention and Protection at Work, 3001 Heverlee, Belgium

**Keywords:** DNA methylation, exposure, endocrine disruptors, farmworkers, human biomonitoring, Morocco, pesticides, risk assessment

## Abstract

Pesticides are compounds of major use in agriculture worldwide. Nevertheless, many pesticide chemicals are classified as endocrine disruptors and potentially carcinogens. Farmers and farmworkers are particularly exposed and are at high risk of developing health-related impairments. In Morocco, the lack of awareness towards pesticide hazards and the inappropriate application of safety measures might increase the exposure as well as the risks of health concerns. In this paper, we present the framework of a study designed to assess pesticide exposure among Moroccan farmers and farmworkers and to evaluate potential health effects, namely endocrine and epigenetic impacts. Human biological monitoring will be conducted to determine pesticide levels in urine following the development and validation of sensitive chromatography methods (SPE, UPLC-MS/MS). Biomarkers of exposure include a set of parent and metabolite pesticide compounds (organophosphates, pyrethroids, triazines and urea-based pesticides). Thyroid and reproductive hormones (TSH, T_3_, T_4_, FSH and LH) as well as global and specific DNA methylation markers (5-mC, 5-hmC, N^6^-mA, THRB and LHR) are selected as biomarkers of effects. This provides guiding steps and methods to perform reliable exposure evaluation and health impact assessment. This study aims to expand the current knowledge on the endocrine and epigenetic risks related to pesticides, especially in low- and middle-income countries.

## 1. Introduction

Farmers and farmworkers are vulnerable to pesticide exposure [1]. High levels of pesticides and their metabolites have been detected in farmers in different regions of the world and have been significantly associated with health concerns such as neural and cognitive disorders [2] and the disruption of endocrine functions [3,4]. In agriculture, pesticides improve yielding by controlling pests as well as plant and livestock disease vectors [5]. Nevertheless, exposure to pesticides, especially among farmers and farmworkers, might lead to adverse health complications that closely involve DNA damage and endocrine alterations [1]. Many pesticides have been identified as endocrine disruptors [6], potentially affecting endocrine functions by mimicking natural hormones and interfering with biochemical processes [7]. Organophosphate pesticides and pyrethroids can be associated with fluctuating levels of thyroid hormones, namely, thyroid-stimulating hormone (TSH), triiodothyronine (T_3_) and thyroxine (T_4_) [8,9]. These categories can also alter the reproductive function, observed mostly in men through fertility reduction [10]. Moreover, pesticides have been significantly related with increased risks of hypothyroidism [11], diabetes [12], obesity [13] and ovarian cancer [14] in farmers.

Epigenetic modifications, namely, DNA methylation, can be a considerable pattern that may explain the occurrence of diseases that include endocrine disruption, such as thyroid cancer [15] and breast cancer [16]. These modifications might mediate the persistent effects of chemicals like pesticides, by influencing gene expression [17,18]. DNA methylation can be involved in mechanisms of either silencing genes, inhibiting the expression of hormone nuclear receptors, or activating growth-promoting genes, which increases the risk of cancer proliferation [19]. Pesticides have widely been associated with endocrine diseases without clear underlying mechanisms; however, the link can be explained by epigenetic modifications, namely, DNA methylation [20].

Exposure to pesticides may occur directly through occupational and household activities or indirectly via contaminated air, food, soil or water [21]. Dermal exposure is commonly important among farmers due to the splashing, spillage or spray drift of pesticides [22,23]. Exposure can also occur via inhalation of volatile compounds present in the air or dust [24]. Nevertheless, oral ingestion of pesticides is likely the most common poisoning route and can lead to serious health effects and to death at low doses [25]. Globally, different safety measures are recommended for pesticide handling [26]. However, these measures are followed inconsistently due to demographic, environmental, psychological, economic and working factors, especially in developing countries where a large group of farmers and farmworkers are unable to afford personal protective equipment (PPE) or do not use them properly [27].

In Morocco, farmers in different regions lack good practices for pesticide application, ignoring potential dangers to health [28]. The purchase of hazardous pesticides, the incorrect management of compound storage and waste, and the lack of PPE are important factors that put Moroccan farmers and farmworkers at significant risk of exposure. Illegal markets for pesticides, unauthorized distribution channels and the misuse of phytosanitary products are additional aspects that enhance the exposure to pesticides and increase the risks to health for farmworkers as well as for the general population in Morocco. This can be further explained by the educational level, the lack of training, farming habits and financial status [29].

This paper presents a study framework aimed at characterizing major changes in endocrine systems (thyroid and reproductive) and in the epigenetic profile (DNA methylation) of Moroccan farmers and farmworkers, as well as atinvestigating possible links with pesticide exposure. As a global goal, this study aspires to increase knowledge on pesticide hazards and enhance awareness on potential health effects.

The subsequent objectives of this study are as follows:

1. Assess farming conditions, pesticide application, protection and health status of farmers and farmworkers in Morocco.
2. Evaluate individual exposure by determining biological levels of pesticide compounds.
3. Study endocrine and epigenetic profiles by determining hormone- and global/gene-specific DNA methylation levels and investigate possible correlations with pesticide exposure.

### 1.1. Pesticide Categories of Interest and Potential Endocrine-Disrupting Effects

#### 1.1.1. Organophosphate Pesticides

Organophosphorus pesticides (OPs) are mostly used to mitigate insects and miticides in agricultural fields [30]. However, compounds of this category can have an antagonistic role and inhibit natural hormone functions in different organs, such as the brain [31]. For instance, malathion disturbs the biosynthesis of thyroid hormones, namely, via the downregulation of the TSH receptor [32]. Chlorpyrifos was also demonstrated to induce thyroid alterations, namely, by decreasing thyroid hormones and increasing thyroid necrotic cells [33]. At the reproductive level, OPs like dimethoate and chlorpyrifos metabolites can significantly reduce sperm quality, increase sperm damage and disturb the mitochondrial function [34]. Although evidence on OPs’ effects in humans is limited, some studies have shown that OPs can induce hormonal fluctuations in farmers by increasing follicle-stimulating hormone (FSH) and luteinizing hormone (LH) levels [35].

#### 1.1.2. Pyrethroids

Pyrethroids are synthetic insecticides based on the naturally occurring pyrethrin pesticides, which are known for their high tendency to kill insects [36]. Pyrethroids are also characterized by their antagonistic action on thyroid hormones and their potential to modulate gene expression, namely genes encoding for the thyroid receptors TRα and TRβ [8]. Pyrethroids and their metabolites have agonist and antagonist effects on androgen and estrogen receptors as well; they can inhibit steroid synthesis and induce oxidative stress [37]. Pyrethroids have largely been detected in humans, even in non-exposed populations, and have been significantly associated with variations in reproductive hormone levels [38]. For example, 3-phenoxybenzoic acid (3-PBA), which is a common pyrethroid metabolite, has been associated with hormonal fluctuations, suggesting a negative correlation with thyroid hormones, namely, the free triiodothyronine (FT_3_) [39], and positive correlations with reproduction hormones, including FSH and LH [40].

#### 1.1.3. Triazines

Triazines are mostly applied in agriculture as herbicides to control unwanted herbs and weeds and are characterized by their broad spectrum, high performance and low cost [41]. Effects of triazines on thyroid function have slightly been investigated. Some experiments have reported increased levels of total triiodothyronine (TT_3_) at some defined doses of atrazine metabolites [42]. On the other hand, the adverse effects of triazines on the reproductive system have been studied more. Atrazine and its metabolites are likely to induce hormone fluctuations, such as reducing the pulsatile release of GnRH [43]. Atrazine and its metabolites can also alter the ovarian function in females [44], inhibit follicle development [45] and reduce testosterone and LHs in males [46]. Other triazines, such as simazine, have also been demonstrated to decrease female puberty and interfere with female hormones [47].

#### 1.1.4. Urea-Based Pesticides

Urea-based pesticides are used against several annual and perennial weeds to control bush and harmful plants in irrigation and drainage ditches [48]. Urea-based herbicides such as linuron can alter thyroid function through abnormal TSH, T_3_ and T_4_ levels [49] and affect the reproductive function by inhibiting estrogen signaling [50]. Diuron, one of the most commonly used urea-based herbicides worldwide, also affects the thyroid hormone profile [51] and inhibits the synthesis of some reproductive hormones [52]. This evidence suggests that urea-based pesticides might have important endocrine-disrupting potential. However, epidemiological data on the human health effects of this category are limited.

### 1.2. Epigenetic Effects of the Target Pesticide Categories

Pesticides have widely been associated with epigenetic modifications, namely, DNA methylation [53]. Organophosphorus pesticides are associated with genome-wide differential DNA methylation [54] and with alterations in DNA methylation levels at gene promoters, which may play a pathological role in disease development [55]. Pyrethroids, namely, permethrin and some metabolites, can induce DNA hypomethylation and can modify gene expression by altering the methylation profile of gene promoters [56]. In the triazine category, atrazine and its metabolites have been linked with DNA hypomethylation in gonads and other tissues and have been shown to alter DNA methyltransferase (DNMT) activity [57,58,59]. Additionally, triazines may contribute to reproductive toxicity through histone and adenine methylation [60]. Although research evidence on the epigenetic or genotoxic effects of urea-based pesticides is limited, some studies have shown that linuron and diuron can significantly induce changes in DNA methylation status. Linuron was shown to be correlated with affected phenotypes in the metabolic and reproductive systems and can lead to the formation of differentially methylated regions (DMRs) in the somatotropic and thyrotropic axes [61]. On the other hand, diuron has been associated with global DNA hypomethylation [62] and with a decrease in total methyltransferase activity [63].

### 1.3. Study Endpoints

#### 1.3.1. Biomarkers of Exposure

**Figure 1:**
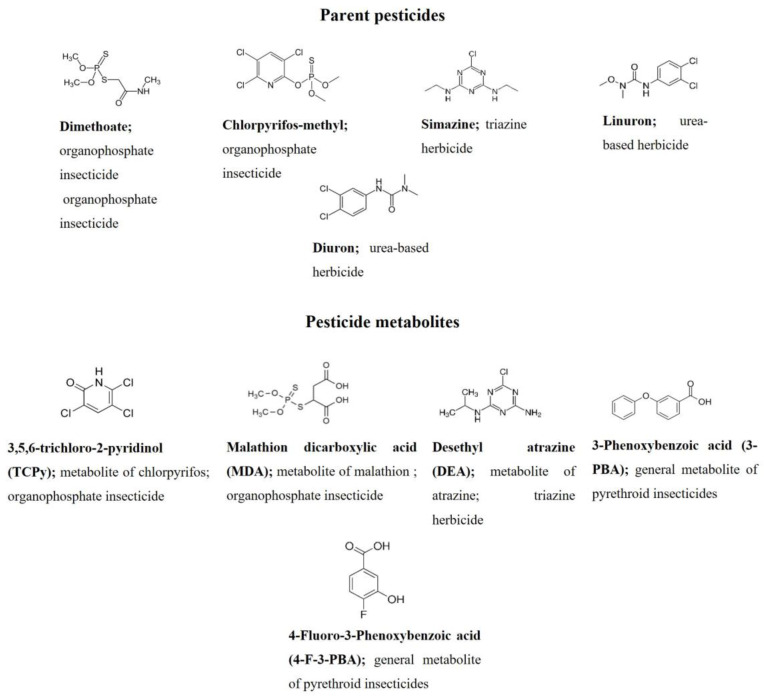
Overview of the targeted pesticide compounds and metabolites.

The pesticide compounds targeted in this study are shown in Figure 1. Parent compounds were selected based on previous ecological and health risk assessment research in the Saïss plane, including Fes–Meknes region. The study of [64] reported that the groundwater near cultivated fields contained a variety of pesticides. Organophosphorus pesticides, namely, chlorpyrifos-methyl, dimethoate and malathion, were the most commonly detected. Urea-based pesticides such as diuron and linuron were among the cumulative pesticides found at concentrations exceeding the limit of quantification. Moreover, pyrethroid levels exceeded the precautionary quality standard limit (0.1 µg. L^−1^) in groundwater, set in the Groundwater Directives of the European Parliament [65]. Triazines, namely, atrazine and its metabolites, as well as simazine, were detected less frequently. The importance of analyzing parent pesticides in farmers in our study consists mainly of investigating human exposure to the same compounds found in the environment of the study area. Additional metabolites, which constitute the major and most common metabolites of the abovementioned pesticide categories, were also considered for exposure assessment in this research. The metabolites of interest include malathion dicarboxylic acid (MDA) (primary metabolite of malathion), 3,5,6-trichloro-2-pyridinol (TCPy) (main metabolite of chlorpyrifos), 3-phenoxybenzoic acid (3-PBA) and 4-fluoro-3-phenoxybenzoic acid (4-F-3-PBA) (common metabolites of pyrethroids), and desethyl atrazine (DEA) (major metabolite of atrazine). Human biomonitoring of pesticide metabolites can indicate long-term exposure or accumulation, and can provide valuable information on potential toxicity and adverse health effects, such as endocrine disruption. In our study, pesticide compounds will be measured in urine, and the levels will be adjusted based on individual creatinine levels to correct potential variations in urine concentration and hydration status of participants.

#### 1.3.2. Biomarkers of Effect

a. Endocrine biomarkers

Thyroid hormones, namely, TSH, T_3_ and T_4_, and the reproductive hormones FSH and LH are selected as endocrine biomarkers in our study. Hormone levels are compared with medical reference values, and correlations are tested with pesticide concentrations and DNA methylation levels.

The thyroid gland plays a crucial role in biological development by maintaining chemical reactions as well as metabolism in the body [66]. By secreting thyroid hormones T_3_ and T_4_, several functions are regulated, such as oxygen consumption, cardiac and respiratory rates, nervous responsiveness, the ovulatory cycle and spermatogenesis [67]. Nevertheless, thyroid hormones are produced only following a regulated release of TSH by the hypothalamic–pituitary axis [68], which might be dysregulated by endocrine-disrupting chemicals. The reproductive system in both men and women is also susceptible to environmental changes and chemical exposure, including pesticides [69]. The functions of reproductive glands are regulated via the normal secretion of FSH and LH, which are responsible for regulating steroidogenesis and gametogenesis [70]. In particular, these hormones are secreted in men to drive testosterone synthesis as well as sperm production and development, and they play important roles in follicle maturation and ovulation in women [71].

b. Epigenetic modifications—global DNA methylation markers

Epigenetic modifications can interact with the endocrine system and may influence hormones involved in development, reproduction and metabolism [72]. Epigenetic markers play an important role in thyroid hormone regulation [73]. Nevertheless, some studies have demonstrated that cytosine methylation can influence iodine uptake in the thyroid gland [72]. Additionally, epigenetic modifications in thyroid-related genes can alter hormonal functioning in different tissues and can lead to adverse health outcomes like cancer [19]. In our study, 5-methylcytosine (5-mC) and 5-hydroxymethylcytosine (5-hmC) are the main markers selected to assess global DNA methylation. 5-mC and 5-hmC are among the most studied epigenetic modifications and play an important role in gene expression and pathogenesis [74]. At the molecular level, 5-mC results from the methylation of the fifth position of cytosine, which is catalyzed by DNA methyltransferases (DNMTs), and it is involved in the regulation of gene transcription as well as in genomic stability [75]. The oxidation of 5-mC generates 5-hmC, which is a stable methylation marker involved in the activation of gene transcription and DNA demethylation [76] Adenine methylation is also investigated in our study through the N^6^-mA marker, for global DNA methylation assessment. Adenine methylation is a novel marker that involves the addition of a methyl group (CH_3_) to the exocyclic NH_2_ at the sixth position of the purine ring in adenine from S-adenosyl-L-methionine (AdoMet/SAM) via the machinery of specific methyltransferase enzymes [77]. N^6^-mA is prevalent in eukaryotes and plays a regulatory role in DNA transcription, transposon activation and stress response [78]. A study by [79] demonstrated that adenine methylation intervenes in the prenatal development and embryogenesis, as well as during fetal differentiation. In humans, N^6^-mA is being newly investigated. It has been detected in children and has been significantly associated with environmental pollutants, namely, heavy metals [80]. Adenine methylation can be involved in some forms of human cancers, such as glioblastoma [81] and ovarian cancer [82]. Nonetheless, its association with health outcomes, including endocrine disruption, has not been investigated, to the best of our knowledge.

c. Epigenetic modifications—gene-specific DNA methylation markers

The thyroid hormone receptor beta (THRB) and luteinizing hormone receptor (LHR) genes are selected in our study to assess specific DNA methylation patterns related to endocrine regulation. THRB encodes thyroid hormone receptor β (THRβ), which plays an important role in the functional regulation of the hypothalamus–pituitary–thyroid system, metabolism and other organs [83]. An altered methylation of THRB might affect gene expression and may lead to adverse health complications, such as metabolic syndrome [84]. The LHR gene encodes luteinizing hormone receptor that binds to LH and GnRH hormones to regulate the production of steroid hormones and maintain pregnancy [85]. This receptor is involved in critical reproductive processes and plays an important role in gonadal steroidogenesis, oocyte maturation, ovulation and male differentiation [86]. DNA methylation of the LHR can be related to reproductive issues, namely, polycystic ovary syndrome (PCOS), which might be associated with decreased methylation of the LHR gene [87].

## 2. Materials and Methods

### 2.1. Study Design

#### 2.1.1. Study Area

**Figure 2:**
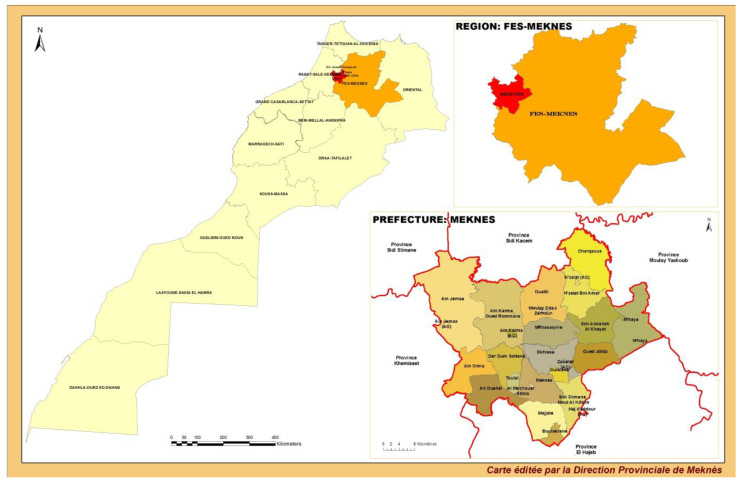
Map of Meknes Prefecture in Morocco showing the study areas of interest. (Meknes Provincial Directorate: https://www.hcp.ma/region-meknes/?preaction=mobile&start=9; accessed on 23 May 2024).

Our study takes place in Meknes Prefecture, Morocco (Figure 2). The Prefecture is located in the Saïss plain in northern Morocco (34° N, 6° W, 546 m) and extends over an area of 1786 km^2^ with an Atlantic climate [88]. This study started in 2022 and is currently being conducted in two main districts of Meknes Prefecture, that are Zerhoun and Aïn-Orma, which include the majority of rural communities. According to the monography of Meknes provided in 2020 by the High Commission for Planning of the Fes-Meknes region, these districts are known for their important and diverse agriculture and comprise approximately 17.7% of the Prefecture’s habitants, being mostly involved in farming and agriculture professions. In the agricultural season of 2019–2020, a total area of 145,100 Ha was cultivated in Meknes Prefecture, resulting in 644,722.3 tons of food products. The main crops in the area are vegetables, fodder, fruit trees, cereals and oil plantations. Farming activity in Meknes Prefecture is considerable and significantly contributes to the agricultural production of the Fes-Meknes region by almost 15.5% [88].

#### 2.1.2. Study Population and Inclusion Criteria

The population of interest in our study consists of farmers and farmworkers. We define a farmer as the person who owns a farmland and engages in agriculture by raising food crops or livestock, while we define a farmworker as the person employed in farm labor activities, including pesticide application and spraying. Farmers and farmworkers can share the same tasks. However, we assume that the workload and the exposure might be greater for farmworkers likely work in several farmlands within one agricultural season. Our cross-sectional study consists of a series of field study campaigns aimed at including two groups of participants (exposed and control individuals) during the spring season. This season is chosen for recruitment since it is the period of large pesticide application in agricultural fields. The exposed group consists of 200 farmers and farmworkers actively involved in farming activities. The control group includes 50 individuals with different professions not being involved in agriculture or activities that may be related with occupational exposure to pesticides. In terms of inclusion, participants from both groups are adult men and women aged between 18 and 65 years, living and working in Meknes Prefecture for at least two years, owning farmland or working in crop fields (for the exposed group), not having any farming activity (for the control group) and being able to consent deliberately to participation.

#### 2.1.3. Recruitment and Ethics

To proceed with recruitment, field study campaigns are organized in accordance with local centers of the National Office for Agriculture Counseling (ONCA) and with the competent health authority services of each of Meknes Prefecture and the districts. For the exposed group, farmers and farmworkers are informed about the study by the local agriculture centers and are invited to thecampaigns. For the control group, the research team investigate private and public institutions in Meknes city and seek age- and sex-matched citizens for voluntary participation. On campaign days, our team members are present onsite to explain the study and answer questions. Participants are requested to approve their participation through an informed consent form (ICF), which is co-signed by either the principal investigator or the research assistant of the study. In adherence with ethical principles and with the Declaration of Helsinki, participants are informed clearly about their role and rights in the study. They provide consent on participation, with possibility for withdrawal at any moment without necessarily giving reasons. Prior to recruitment, our study obtained ethical approval (No 21 Mar 2021) from the Ethics Committee for Biomedical Research (CERB) of Moulay Ismail University of Meknes in Morocco. Additionally, a Material Transfer Agreement (MTA) was established, including all involved institutions and laboratories, for any required data or sample transfer.

### 2.2. Data Collection and Sampling Procedure

#### 2.2.1. Study Survey

During the study campaigns, participants are asked to fill out a harmonized survey, introduced and completed with the help of our research team. The data being collected concern living, occupational and health aspects. In the first part of the survey, participants are requested to provide anthropometric data, including sex, age, weight and height. Then, they are asked about demographic aspects, namely, the living area, education and income. The second part of the survey includes questions on work frequency, lifestyle and suspected risk factors related to pesticide exposure, namely the use of PPE. Additional questions about farming tasks and work history are also included. This is to gather comprehensive information on farm fields, pesticide application, pesticide storage and waste, in addition to personal protection. The last section of the survey is dedicated to assessing acts related to hygiene and the health status of participants. To ensure the confidentiality, we opt for pseudonymization of the data in the processing and analysis. During recruitment, participants are aware that data are collected and analyzed for research purposes only, without any communication or transfer to third parties not involved in the study. Our survey is inspired by the Survey on PEstiCIde Mixtures in Europe (SPECIMEn) aimed at generating new pesticide exposure data in a harmonized European setting, as part of the European Human Biomonitoring Initiative HBM4EU [89].

#### 2.2.2. Sampling of Biological Matrices

**Figure 3:**
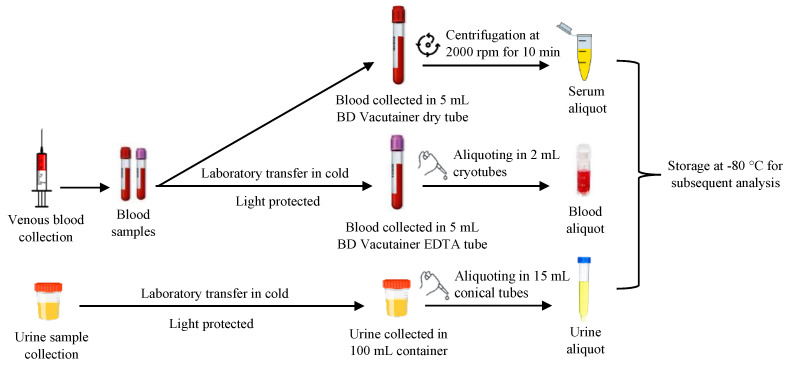
Procedure for biological matrix collection and processing.

Along with the survey, participants are asked to provide urine and blood samples to be used for medical, biomonitoring and epigenetic analysis. The sampling procedure is shown in Figure 3. Logistics are prepared beforehand to ensure a good conduct of the sampling campaigns and a secure transfer of samples for lab analysis. Briefly, two samples of venous blood are collected in 5 mL BD Vacutainer tubes, in addition to one urine sample collected in a 100 mL sterile urine container. After collection, samples are immediately transferred to the laboratory under cold conditions (within a maximum of 4 h) for processing, aliquoting and storage. The first blood sample is collected in a dry tube and centrifuged at 2000 rpm for 10 min. This sample generates serum that will be used in medical analysis for hormone assessment. The second blood sample is collected in an EDTA tube, to be used for epigenetic analysis. At the end of the process, all the collected samples are aliquoted and stored at −80 °C until analysis. In this study, we carefully consider the safety of participants and we ensure that risks and harms related to the sampling are reduced to the lowest possible. This is ensured by the presence of a nursing team that assists participants, mainly during the sampling. In case of any health complications, medical assistance is provided.

## 3. Analytical Design

### 3.1. Analytical Methods for Exposure and Health Effect Biomarker Assessment

#### 3.1.1. Method Development and Validation Process for Human Biomonitoring Analysis of Pesticides

The selected pesticide compounds and metabolites will be analyzed in urine via chromatography techniques. Therefore, Ultra-Performance Liquid Chromatography coupled with tandem Mass Spectrometry (UPLC–MS/MS) and Solid-Phase Extraction (SPE) methods are being developed and validated, respectively, for reliable biological monitoring.

The UPLC–MS/MS method is set in place using Multiple Reaction Monitoring (MRM) for high sensitivity and selectivity in compound identification and quantification, namely, through the selection of specific mass transitions. This method is being optimized for adequate mass spectrometry (MS) parameters, such as ionization mode, operating mode, ionization voltage, collision energy, ion source temperature, the resolution over which ions are detected and distinguished and signal-to-noise ratio (S/N). Liquid chromatography (LC) conditions are established and adapted to obtain ideal separation of the compounds with an optimal retention time by selecting the type and dimensions of the LC column, mobile phase composition (water, acetonitrile, methanol) and additives (ammonium formate, formic acid, acetic acid), gradient, flow rate and injection volume. This is achieved by injecting relatively high concentrations of standard compounds (50–100 ng. mL^−1^) prepared from stock and working solutions.

The SPE technique is used to extract pesticides from urine. Our method is based on the work of [90]. Briefly, urine is prepared by adding appropriate volumes of internal standards of pesticide chemicals and the β-glucuronidase enzyme for the hydrolysis of glucuronide conjugates, besides a set of reagents like formic acid and sodium acetate buffer. Following the preparation step, urine samples are incubated at 37 °C for 2 h. The extraction phase consists of using Oasis HLB cartridges (60 mg, 3 cc, Waters, Milford, MA, USA), based on a hydrophilic–lipophilic balance. Extraction steps start with conditioning the cartridges with a set of solvents (dichloromethane, methanol and water), then loading samples, washing with a solution of water and methanol (95:5; *v*/*v*) and eluting the compounds of interest with methanol. Extracts are evaporated at 32 °C until approximately 1 mL is left, then 50 µL of water is added to avoid complete evaporation. In our study, the different steps of this extraction method are being modified and optimized to be more adequate to our target pesticides and to increase the extraction efficiency. To this end, we are working on determining new validation parameters following the changes being made to the initial method. This mainly consists of defining the limits of detection (LLOD) and quantification (LLOQ), the analytical calibration range, linearity, specificity, precision, accuracy, extraction efficiency and matrix effect.

#### 3.1.2. Process for Endocrine and DNA Methylation Biomarker Analysis

Endocrine markers, namely, thyroid and reproductive hormones, will be measured in a medical testing laboratory via routine analytical methods, no later than 1 month after sample collection. Global and gene-specific DNA methylation markers will be measured via UPLC–MS/MS and pyrosequencing, respectively.

For epigenetic analysis, DNA will be extracted from blood samples using the QIAmp^®^ Blood Mini Kit (Qiagen, Hilden, Germany), followed by the determination of DNA purity and recovery via spectrophotometry. For global DNA methylation assessment, DNA will be hydrolyzed into deoxyribonucleosides to determine levels of methylation markers via UPLC–MS/MS, following the analytical procedure described in the study of [91]. Cytosine methylation and hydroxymethylation, as well as adenine methylation can be expressed as follows:

- % Cytosine methylation = [5-mC]/[5-mC + 5-hmC + 2-dC]
- % Cytosine hydroxymethylation = [5-hmC]/[5-mC + 5-hmC + 2-dC]
- % Adenine methylation = [N^6^-mA]/[2-dA + N^6^-mA]

Specific DNA methylation of the THRB and LHR genes will be assessed by bisulfate conversion and pyrosequencing following the analytical method described in the study of [91]. Briefly, bisulfate conversion will be applied to DNA extracts to convert unmethylated cytosine into uracil. The promoter regions of genes will be amplified via polymerase chain reaction (PCR) using appropriate primers. Then, a pyrosequencing reaction will be performed to define the nucleotide content of the amplicons and determine their methylation levels.

### 3.2. Data Analysis

Data from the different analysis will be statistically processed using R software (v4.1.2; R Core Team 2022). Survey data will be analyzed to describe participant characteristics as well as factors that might enhance their exposure to pesticides or affect their health. Biomonitoring data will be explored for the urinary levels of pesticide compounds, following the necessary adjustments to creatinine levels. Quantifiable pesticide concentrations will be compared to levels in the literature and to limit values established by environmental agencies or legislations, if available, to evaluate the extent of exposure. Endocrine and epigenetic data will be tested based on the hormone and DNA methylation levels, that will help in detecting potential abnormalities. The data distribution will be evaluated using the Shapiro–Wilk test. Quantitative values will be compared between exposed and non-exposed groups based on the arithmetic mean (+/− standard deviation) via a *t* test, whereas qualitative data will be analyzed via the parametric or non-parametric tests of ANOVA and Kruskal–Wallis, depending on distribution normality. Correlations using linear models will be performed by defining dependent and independent variables and by using Pearson’s or Spearman’s test depending on the data distributions. Major correlations will be conducted for quantitative data, considering endocrine and DNA methylation levels in addition to pesticide values above the LOD. In this study, 0.05 is defined as the conventional *p*-value to consider the results statistically significant.

## 4. Expected Results

Farmers and farmworkers are often exposed to pesticides in their work field. This can potentially disturb their endocrine system by interfering with hormone production, regulation and receptor function, and can likely affect their epigenetic profile, namely, through DNA methylation modifications. Considering the important workload of farmers and farmworkers in Morocco and the lack of protection measures, biomonitoring findings might reveal relatively high levels of pesticides and metabolites. Medical and epigenetic analysis may uncover less balanced levels of thyroid and reproductive hormones, in addition to fluctuating levels of DNA methylation markers, which could indicate long-term alterations in gene expression. Consequently, the results from this study are expected to reveal significant correlations between pesticide exposure and hormonal disparities as well as DNA methylation modifications. The results are also expected to provide insights on potential mechanisms involved in endocrine disruption and DNA methylation patterns related to pesticide exposure. Moreover, we aspire to demonstrate, through an explanatory approach, the mediating role that global and specific DNA methylation might play in enhancing disparities of hormone levels in the context of pesticide exposure.

Overall, we have the ambition to use the findings of this study to provide recent insights about the occupational situation of farmers and farmworkers in Morocco. This involves drawing up recommendations for safer agricultural practices and insisting on the importance of incorporating protection guidelines and measures in agricultural work. This is ultimately aimed at improving the working conditions of farmers and farmworkers and protecting their health in Morocco.

## 5. Scope and Strengths of the Study

This scientific study represents a groundbreaking effort in assessing the endocrine and epigenetic effects of pesticides in Morocco. Given the widespread use of pesticides in agriculture and their potential health risks, understanding their impact on hormonal balance and gene expression is crucial. This research fills a significant gap in the scientific literature by providing original data specific to Morocco’s environmental and agricultural context, and will contribute to global discussions on pesticide safety, aimed at mitigating potential risks to human health and ecosystems. Specifically, this cross-sectional study targets farmers and farmworkers as the population of interest, being at greater risk of exposure to pesticides and their health impacts.Targeting two groups of exposed and non-exposed individuals supports a solid analytical approach, demonstrating potential differences in individual exposure.

This study combines chemical and biological measurements for a reliable assessment. In biomonitoring analysis, urine is selected to determine the levels of parent pesticides and metabolites. It is a non-invasive, straightforward and easy-to-collect matrix. It can also allow sensitive detection of weakly concentrated chemicals and can indicate recent, acute or chronic exposure to long-lived or cumulative compounds. Blood is chosen for the specific measurement of endocrine and epigenetic biomarkers. Although invasive, blood can provide accurate levels of circulating hormones and can increase DNA recovery, which is essential for DNA methylation assays. Determining hormone levels is highly interesting to study disparities in the endocrine system and investigate possible biological outcomes that might follow. Additionally, DNA methylation levels can provide a global overview on the epigenetic profile of farmers and farmworkers and can be used to explain the potential of pesticides in inducing endocrine-disrupting effects. Risks related to the sampling are limited compared to the future benefits for health that this study can bring for farmers and farmworkers. Insights from this study will lead to important scientific advancements on exposure to pesticides and will allow a comprehensive understanding of its health impacts. Moreover, our findings will contribute to reducing risks of diseases by promoting exposure prevention and health protection, namely, through the improvement of working conditions in farm settings, particularly in low- and middle-income countries.

In this study, we assess the correlations between exposure and health effect biomarkers and we combine survey data with biological data. Information gathered on lifestyle, pesticide use and health status will improve the quality of the data and will contextualize the exposure. This approach will enhance the comprehensive understanding of the sources and pathways of exposure.

Our study is a continuation of the research of other projects initiated in Morocco to assess the impacts of pesticides on health and the environment [92,93,94]. Our research further highlights the relationship between chemical exposure and health risks. Our insights will contribute to preventing endocrine diseases and help explain their occurrence in the context of pesticide application, especially in developing countries. In this sense, we aim to promote the establishment of safe farming practices and to raise awareness on the health risks in occupational activities that include pesticide handling.

## 6. Study Limitations

In Morocco, the actual census of workers in agricultural fields is not available and is not accurately updated, which makes the definition of the total number of farmers and farmworkers in the geographical area of our study difficult. Consequently, this might influence the sample size and may limit data segmentation as well as statistical tests. Nevertheless, the results will be interpreted considering the conventional *p*-value and correlation coefficients. Additionally, statistical adjustments such as Bonferroni correction will be applied if necessary. In terms of exposure and health risk assessment, factors related to exposure, such as the living environment, habits, lifestyle and health conditions, might be specific to participants from Meknes Prefecture, which may limit the extrapolation of the results to other regions of Morocco. Nonetheless, comparisons consider not only quantitative values but also qualitative data for a contextualized interpretation of the results.

## 7. Conclusions

Our study framework describes the main steps to explore pesticide exposure from the source and to comprehensively understand the potential impacts on the endocrine and epigenetic profiles of farmers and farmworkers. This is specifically assessed by measuring endocrine and epigenetic biomarkers, namely, thyroid and reproductive hormones, as well as markers of global and gene-specific DNA methylation. This study also highlights the role of human biomonitoring approach in occupational health by determining the factors of exposure and its extent, which will contribute to defining prevention measures and practices to protect workers’ health. Through this study, we aspire to promote the standardization of the human biomonitoring approach and harmonize exposome and epigenome investigations for better comparability and accuratedata communication, especially in low- and middle-income countries, namely, Morocco, where pesticide use is inevitable.

## Data Availability

The data presented in this study are available on request from the corresponding author.
